# Astrocyte morphology, heterogeneity, and density in the developing African giant rat (*Cricetomys gambianus*)

**DOI:** 10.3389/fnana.2015.00067

**Published:** 2015-05-26

**Authors:** Matthew A. Olude, Oluwaseun A. Mustapha, Oluwatunde A. Aderounmu, James O. Olopade, Amadi O. Ihunwo

**Affiliations:** ^1^Neuroscience Unit, Department of Veterinary Anatomy, Federal University of AgricultureAbeokuta, Nigeria; ^2^Neuroscience Unit, Department of Veterinary Anatomy, University of IbadanIbadan, Nigeria; ^3^School of Anatomical Sciences, Neuroscience Unit, University of the WitwatersrandJohannesburg, South Africa

**Keywords:** GFAP, Golgi stain, astrocyte domains, astrocyte subtypes, African giant rat

## Abstract

Astrocyte morphologies and heterogeneity were described in male African giant rats (AGR; *Cricetomys gambianus*, Waterhouse) across three age groups (five neonates, five juveniles, and five adults) using Silver impregnation method and immunohistochemistry against glial fibrillary acidic protein. Immunopositive cell signaling, cell size and population were least in neonates, followed by adults and juveniles, respectively. In neonates, astrocyte processes were mostly detected within the glia limitans of the mid and hind brain; their cell bodies measuring 32 ± 4.8 μm in diameter against 91 ± 5.4 μm and 75 ± 1.9 μm in juveniles and adults, respectively. Astrocyte heterogeneity in juvenile and adult groups revealed eight subtypes to include fibrous astrocytes chiefly in the corpus callosum and brain stem, protoplasmic astrocytes in the cortex and dentate gyrus (DG); radial glia were found along the olfactory bulb (OB) and subventricular zone (SVZ); velate astrocytes were mainly found in the cerebellum and hippocampus; marginal astrocytes close to the pia mater; Bergmann glia in the molecular layer of the cerebellum; perivascular and periventricular astrocytes in the cortex and third ventricle, respectively. Cell counts from twelve anatomical regions of the brain were significantly higher in juveniles than in adults (*p* ≤ 0.01) using unpaired student *t*-test in the cerebral cortex, pia, corpus callosum, rostral migratory stream, DG, and cerebellum. Highest astrocyte count was found in the DG, while the least count was in the brain stem and sub cortex. Astrocytes along the periventricular layer of the OB are believed to be part of the radial glia system that transport newly formed cells towards the hippocampus and play roles in neurogenesis migration and homeostasis in the AGR. Therefore, astrocyte heterogeneity was examined across age groups in the AGR to determine whether age influences astrocytes population in different regions of the AGR brain and discuss possible functional roles.

## Introduction

The mammalian central nervous system (CNS) had been typically characterized with a neuronal bias. However, other neurocellular components of the CNS such as astrocytes are now known to comprise morphologically diverse and functionally important cells crucial in maintaining homeostasis and protection (Emsley et al., 2004; Nedergaard and Verkhratsky, 2012; Ota et al., 2013). In the early 1900s, astrocytes were grouped into two main sub-types, i.e., protoplasmic or fibrous astrocytes on the basis of differences in their cellular morphologies, anatomical locations, and function. At that time, astrocytes were thought to have only physical structural roles (Ramón y Cajal, 1909, see review by Kettenmann and Ransom, 2005). Scientific advancements now reveal astrocytes as responsible for a wide variety of complex and essential functions in health and neurodegeneration including: buffering glycogen fuel reserves and supporting brain metabolism (Panov et al., 2014); regulation of ion concentration in the extracellular space (Gabriel et al., 2004); promotion of the myelinating activity of oligodendrocytes (Ishibashi et al., 2006; Lutz et al., 2009); formation, maintenance, and pruning of synapses during development and in the adult CNS (Christopherson et al., 2005; Stevens et al., 2007; Barres, 2008); primary roles in synaptic transmission and information processing (Newman, 2003); formation of the gap junctions (Batter et al., 1992; Venance et al., 1998; Sofroniew and Vinters, 2010); regulation of intracellular calcium concentration (Verkhratsky and Kettenman, 1996; Sofroniew and Vinters, 2010); co-ordination of vasomodulation in response to changes in neuronal activity (Takano et al., 2006; Koehler et al., 2009; Quaegebeur et al., 2011); scar formation (Sofroniew and Vinters, 2010) among many others.

Though these findings have elucidated the homeostatic functions of astrocytes in the CNS (Allen et al., 2011), modern techniques have not been able to solve the basic astrocyte complexity particularly with respect to the concept of heterogeneity occurring in several anatomical regions (Hewett, 2009). Thus, the characterization of these diverse cells continually poses a challenge in neurobiology (Kimelberg, 2004). These cells are being progressively demonstrated as having different functional pathways in health and neuropathologies and as such, may affect drug targeting and specific disease treatment (Nedergaard and Verkhratsky, 2012). Therefore, for the functional aspects of astrocytes to make complete sense, heterogeneity has to be properly defined and in particular, in the animal models being used for various studies. A better understanding of astrocyte heterogeneity, will greatly aid investigation of astrocytes functions in the normal brain as well as their roles in neurological disorders. This may potentially reveal neuropathological selectivity, genetic and geographical domains as in the case of adult neurogenesis (von Bohlen and Halbach, 2011; Olude et al., 2014a).

The AGR has been trained to detect landmines and tuberculosis samples using olfactory and cognitive cues (Weetjens et al., 2009; Olude et al., 2014a,b). In our previous study (Olude et al., 2014a), the granule and periventricular cell layers of the AGR OB were shown to contain numerous astrocytes. These are believed to be part of the radial glia of the RMS that aid in tangential migration of newly generated cells from the SVZ to the glomerular layer (where first synaptic transmissions occur) and have an influence on the keen sense of olfaction in this rodent (Kálmán and Hajos, 1989; Kosaka and Kosaka, 2009; Olude et al., 2014b). Similar functional relevance was described in mice when a loss of adenomatous polyposis coli in Bergmann glia with increasing age, disrupted their unique architecture and led to cell non-autonomous neurodegeneration of cerebellar Purkinje neurons (Wang et al., 2011). Furthermore, increase in gray matter in the first few years of life is due largely to glia cell proliferation, dendritic, and axonal arborization and synapse formation (Mrzljak et al., 1991). This proliferation and eventual pruning is believed to be essential for the fine tuning of functional networks of brain tissue in development (Zhang et al., 2006). The AGR, through development demonstrates this pattern of gray matter proliferation but the astrocyte input has not been studied (Olude, 2014). Thus, this study seeks to describe the immunohistochemical localization of astrocytes in the AGR brain across three age groups in order to investigate regional heterogeneity, characterize differences in astrocyte morphology of the AGR brain at defined regions, quantify astrocyte density, adduce possible functional roles and add to the body of knowledge on the brain of the AGR.

## Materials and Methods

### Animals (Sampling, Perfusion, and Brain Harvest)

This study was approved by the Ethics Committee of the University of Ibadan. A total of 15 male African giant rats (*Cricetomys gambianus*), neonates (*n* = 5), juveniles (*n* = 5), and adults (*n* = 5); between neonatal day zero and over 52 weeks with body weights ranging from 20 g to 1150 g were used in this study. The animals were obtained from local hunters in the wild, excluded for head or nervous deformities and stabilized within 24 h. All animals were sedated with a lethal dose of chloroform and were perfused transcardially, first with normal saline followed by 4% paraformaldehyde in 0.1 M phosphate-buffered saline (PBS), pH 7.4. Brains were removed shortly after perfusion, weighed, post-fixed in 4% paraformaldehyde and cryo-protected with 30% sucrose for 2–3 days.

### Golgi Stain Procedures

From one brain, thick sections of 1 cm × 1 cm sections were taken from the neocortex and processed for Golgi Silver impregnation method for neurons. Silver impregnation (Golgi staining) was carried out as described briefly: tissue was fixed in a 3% potassium bichromate (60 ml), 10% formalin (20 ml), 2% silver nitrate solution in the dark for 7 days and solution changed with a fresh solution daily. The tissue blocks were then transferred into 2% silver nitrate solution for 3 days at room temperature in the dark. Filter paper was used to absorb excess solution before putting blocks into silver nitrate solution. The silver nitrate solution (less than regular use) was changed several times until brown precipitates disappeared. Sections were cut at 60 μm thick into distilled water. The sections were mounted on to Superfrost Plus® slides and air-dried for 10 min. These were later dehydrated through 95 and 100% alcohol, cleared in xylene, and cover-slipped.

### Immunohistochemistry

Brains tissues were allowed to equilibrate in 30% sucrose in 0.1 M PB at 40°C. Sagittal sections were taken in series from tissues frozen in 30% sucrose and sectioned into 40 μm thick sections on a freezing microtome (Hyrax®). The immunostaining was performed using the free floating method. Sections were then stained with cresyl violet (for Nissl bodies) to determine anatomical orientation and glial fibrillary acidic protein (GFAP) was used as immunohistochemical marker for astrocytes. One in every 20th section (Adult and Juvenile brains) and one in ten section (for neonates) were utilized and other sections used as control as needed. Sections were washed twice for 10 min in PBS and then rinsed with Tris-Base Saline in Triton (TBST) once for 5 min under gentle shaking at room temperature. Sections were then treated with blocking solution, 5 % normal rabbit serum (NRbS) in TBST for 30 min.

Tissues were then transferred into primary antibody GFAP (Santa Cruz 1:250) in TBST supplemented with 2% bovine serum albumin (BSA) and 2% NRbS overnight at 4°C under gentle shaking. The next day, tissues equilibrated at room temperature followed by a three times 10 min wash in TBST under gentle shaking at room temperature. Secondary antibody, biotinylated rabbit-anti-goat (Vector lab, CA, USA; 1:250) in Tris-Base Saline (TBS) supplemented with 2% NRbS for 60 min at room temperature under gentle shaking was then applied. Sections were then washed in TBS thrice for 10 min 157 each under gentle shaking at room temperature. Thirty minutes prior to the time of use, Avidin Biotin Complex (ABC) reagent in TBS (1:100, A plus B) was applied to the tissues for 40 min at room temperature under gentle shaking. Sections were then washed in TBS twice at 15 min each under gentle shaking at room temperature followed by washing in Tris Base (TB) pH 7.6 two times for 10 min each. Sections were then pre-incubated in DAB solution, 0.5 mg/mL in TB (pH 7.6) by using 2 mL per vial for 30 min in the dark under gentle shaking at room temperature. Thirty-five microliter of 0.5% H_2_O_2_ was added to each vial and mixed adequately and allowed to develop under visual guidance until strong nuclear staining appears within the rostral migratory stream (RMS). The reaction was stopped by washing sections in TB pH 7.6, three times for 10 min each under gentle shaking at room temperature. Tissues were washed in PB, counterstained and mounted onto 0.5% gelatinized slides and air dried overnight. Tissues were then dehydrated in an ascending graded series of alcohol, cleared in xylene and cover slipped with Entelan®. Astrocyte density was defined as the number of cells per mm^2^ in a 40 μm section following the pattern of Emsley and Macklis (2006). Sections of the brains sections were measured, analyzed and photomicrographed with a light microscope using the Zeiss Axioskop 2 plus microscope (Germany). GFAP signaling was quantified using Image J software application (1.46r version). Data was expressed as mean ± SEM and analyzed with SPSS-16 version using one-way ANOVA for soma density across age groups and unpaired “two-tailed” student *t*-test for astrocyte counts between juvenile and adult AGR (*p* ≤ 0.01).

## Results

The precise age of animals used in this study could not be confirmed (since they were captured from the wild) except the neonates which were day old. The age group classification was judged by already reported parameters which include their weight and sexual maturity (Ajayi, 1974); using weight estimates to classify the AGR as neonates (0-70 g; day 0–28), juveniles (over 70 g but below 500 g; day 28–180) and adult (above 500 g, day 180 and above). Mean brain and body weights were given as: neonates = 0.40 ± 0.00 g, 18.68 ± 0.69 g; Juveniles = 4.48 ± 0.18 g, 300 ± 36.51 g, and Adults = 5.48 ± 0.23 g, 975 ± 40.31 g, respectively.

### Morphology of Golgi Stained Astrocytes

**FIGURE 1:**
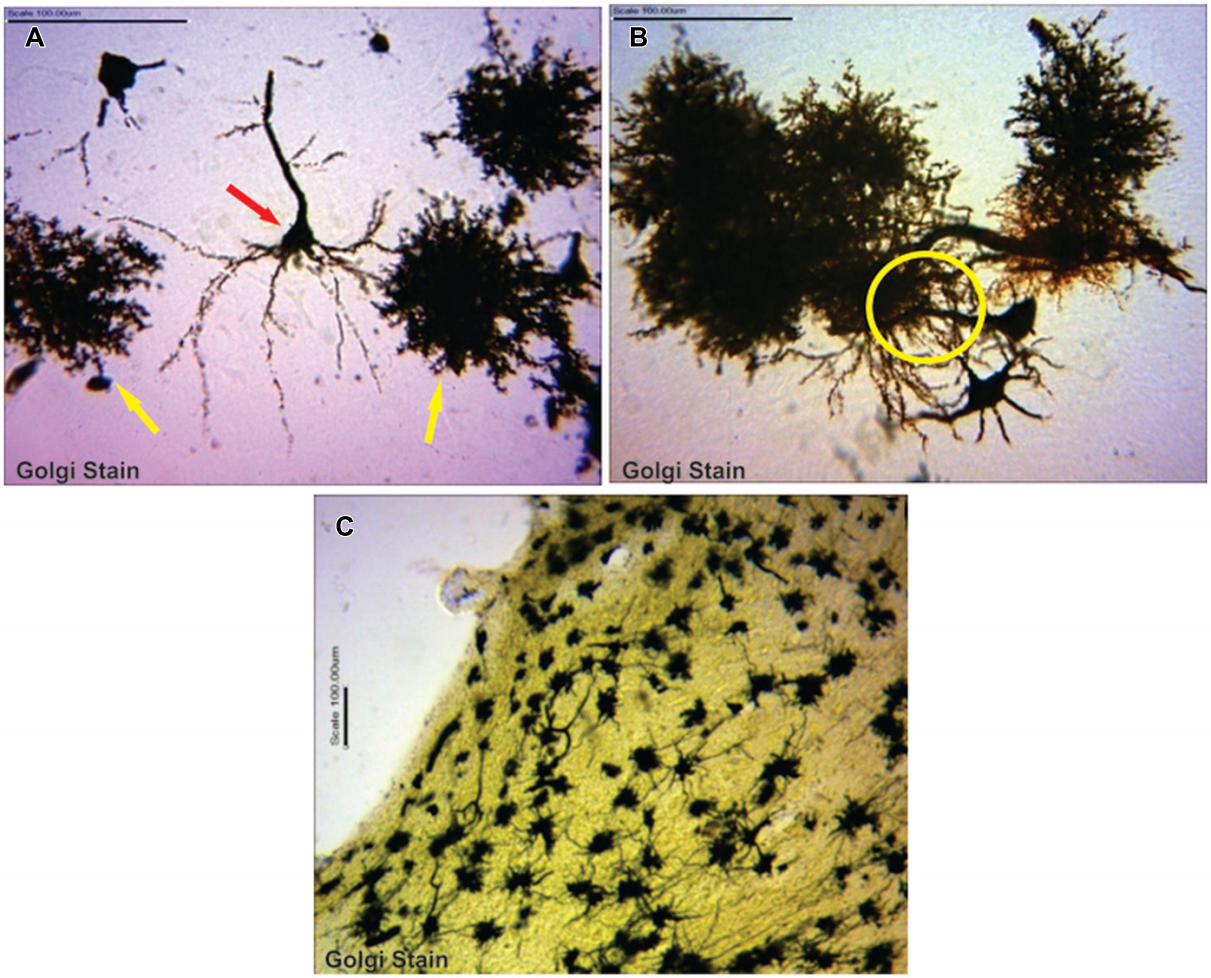
**(A)** Typical pyramidal neuron (red arrow) making association with astrocytes (yellow arrows; **B**) astrocytes in synaptic association with neurons (circumscribed area; **C**) astrocytes immunolabelling in the cerebellum.

Astrocytes were typical, appearing as tufted clumps with perivascular, Bergmann, protoplasmic, and fibrous types readily identified. Also, Golgi staining revealed astrocytic-neuron connections in the cerebral cortex layers (II, III, and V) and, molecular and Purkinje cell layers including the white mater components of the cerebellum (**Figure 1**).

### Age Affects GFAP Signaling Intensity

**FIGURE 2:**
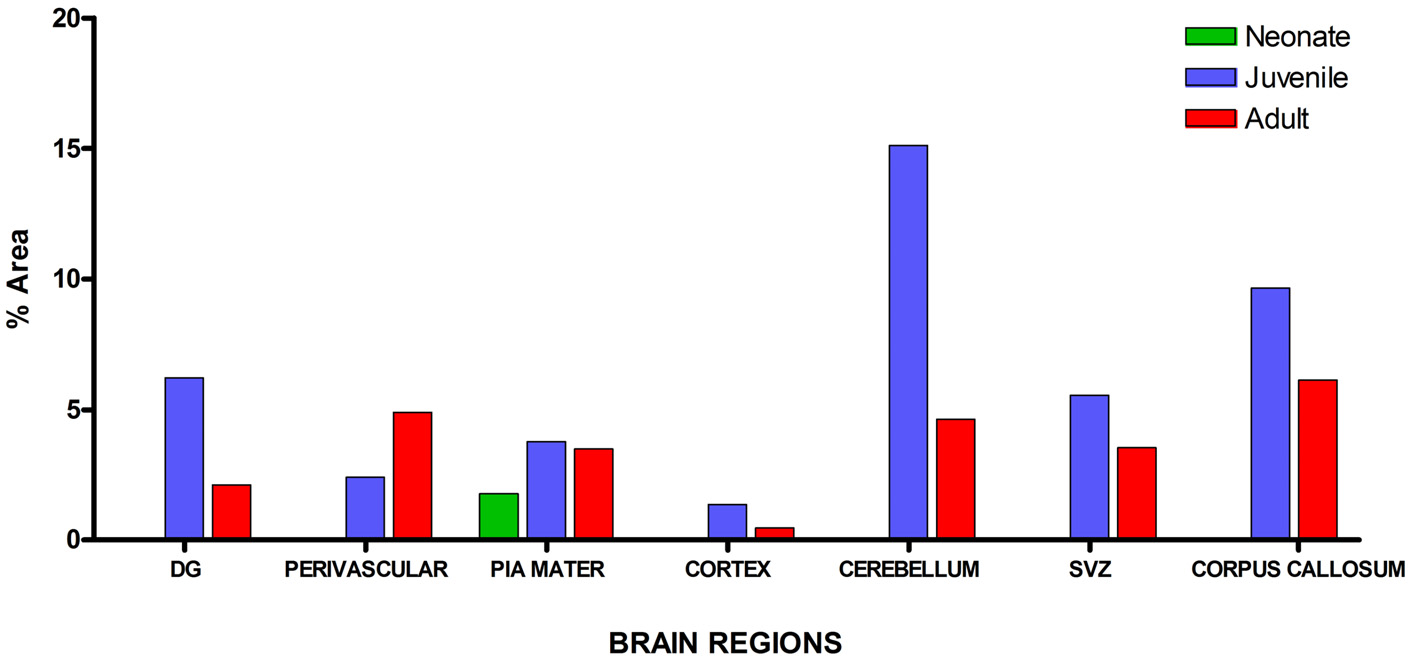
**Quantitative analysis of glial fibrillary acidic protein (GFAP) signal intensity in selected brain regions across age groups revealed greatest signal values per unit area in juveniles followed by adults and neonates, respectively**.

**FIGURE 3:**
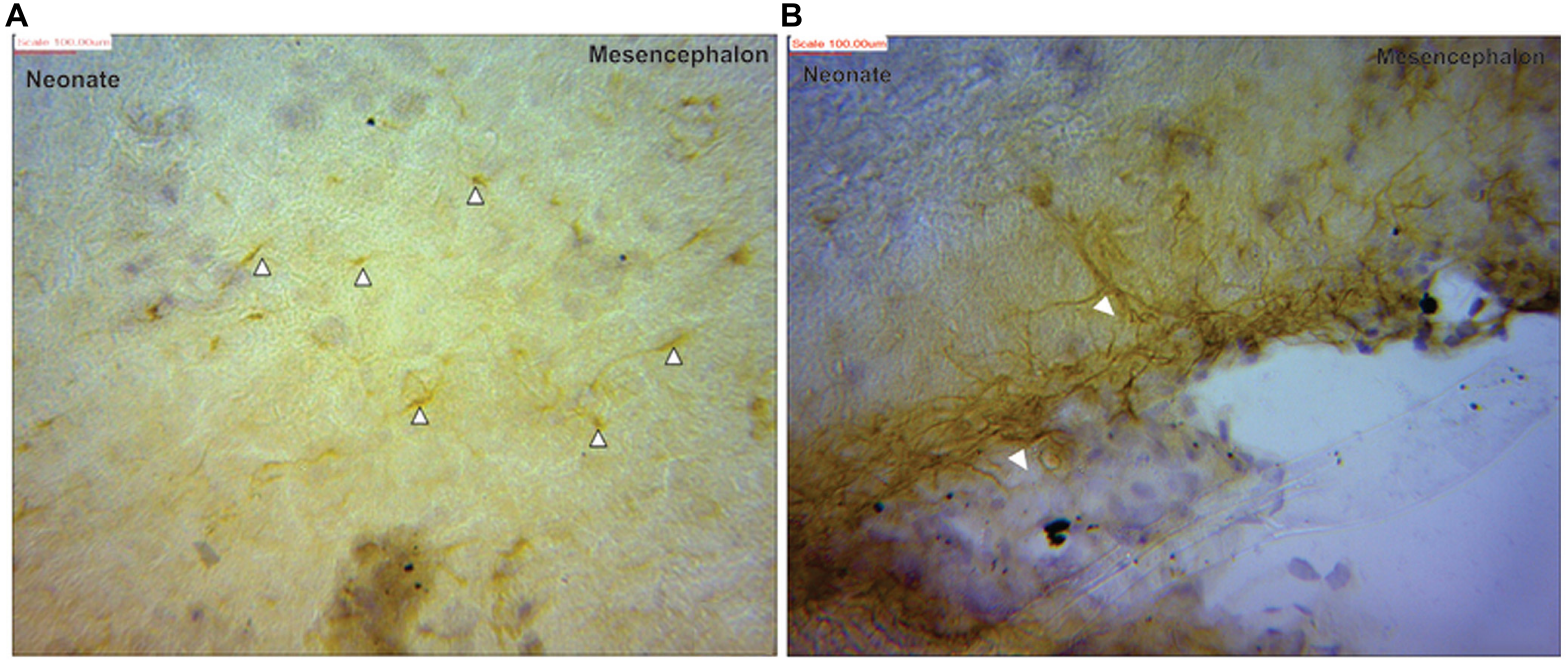
**Glial fibrillary acidic protein positive immunostaining for astrocytes (black arrows) in neonate **(A)** mesencephalon showing more processes than cell bodies **(B)** at the pial surface of the mesencephalic region**.

**FIGURE 4:**
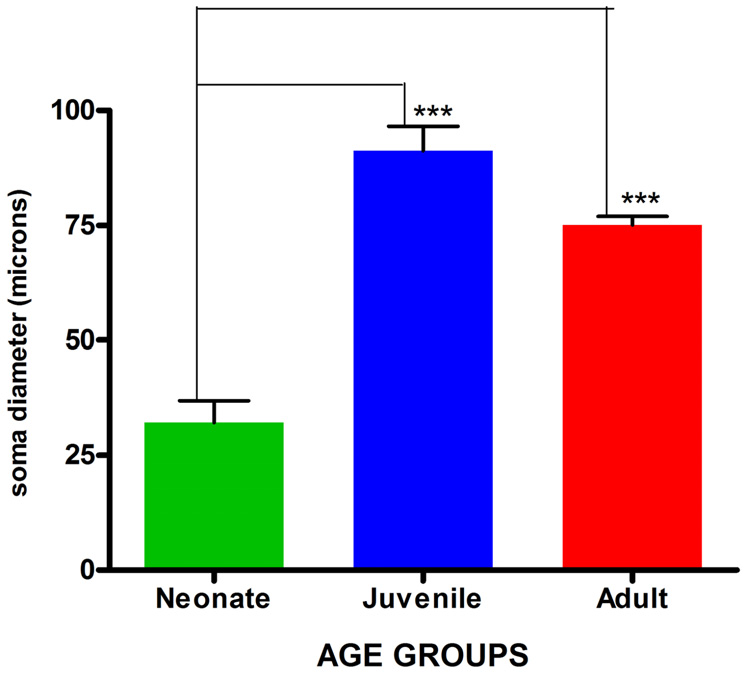
**Comparison of mean astrocytic soma diameters across age groups.** Values are expressed as means ± SEM. ^∗∗∗^indicates significant statistical difference at *p* ≤ 0.01.

Visualization and image J quantification of GFAP astrocytes revealed greatest signaling intensity in juveniles followed by adults and lastly neonates despite using the same dilution factors for staining all sections (**Figure 2**). The pattern of GFAP signaling intensity was caudo-rostral in all the groups. In neonates, processes were more apparent than cell bodies (**Figure 3**). Their cell bodies measured approximately 32 ± 4.8 μm in diameter against 91 ± 5.4 μm and 75 ± 1.9 μm in juveniles and adults, respectively (**Figure 4**). Over 90% of astrocytic cells detected in neonates were within the glia limitans of the germinal zone of the ventriclular region, dorsal, and ventral regions of the mid and hind brain, mesencephalon, and a thin line extending along the hippocampal fissure. The expression of GFAP in the forebrain was confined to a thin line of signal extending along the hippocampal fissure and surrounding the ventricular region. Generally, low levels of signal were observed in the forebrain glia limitans and within the corpus callosum.

**FIGURE 5:**
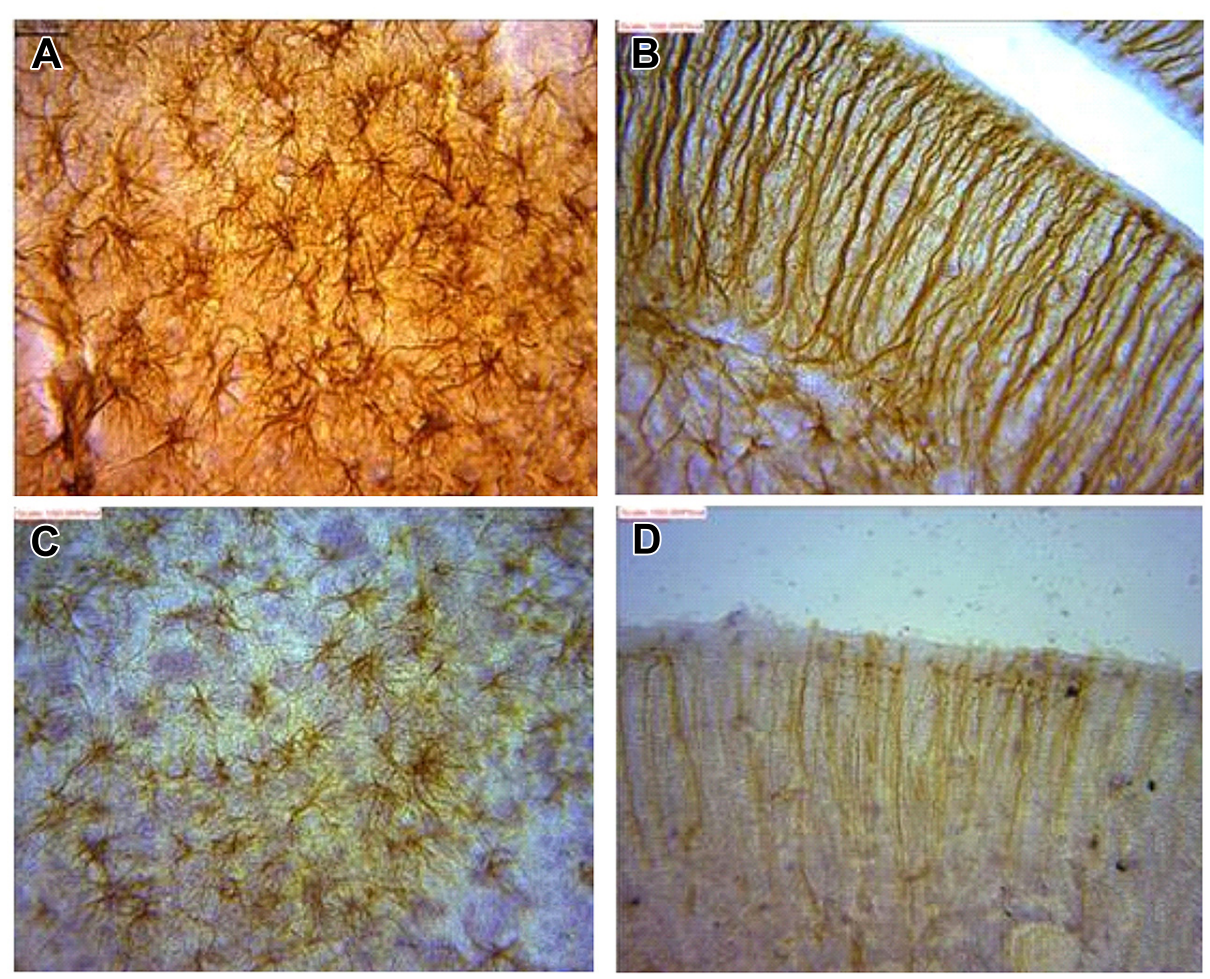
**Astrocytes in **(A)** dentate gyrus (DG) and **(B)** cerebellum of juvenile African giant rats (AGR) showing greater intensity of GFAP signaling as against corresponding areas of adults AGR (C,D)**.

It was observed that immunopositive labeling and the intensity of staining extended rostrally from mesencephalic region to the superficial regions of the cerebellum in the juvenile. This superficial labeling could be localized to the Purkinje cell and remained at moderate and reduced levels in the adult. High levels of GFAP intensity in the glia limitans of these regions had highest intensity in juvenile with reduced expression in adults. GFAP intensity in the juvenile was observed to reach apparent peak levels of expression in this group along the hippocampal area which was more prominent. This pattern decreased to more moderate levels in the adult. The appearance of strong signal within the polymorphic layer of the DG was found in juvenile groups and was similarly reduced in the adult. Similar signal intensity was observed in the cerebellum and hippocampal regions a feature that reduced in adult sections (**Figure 5**).

### Regional Morphological Heterogeneity in the AGR Brain

**FIGURE 6:**
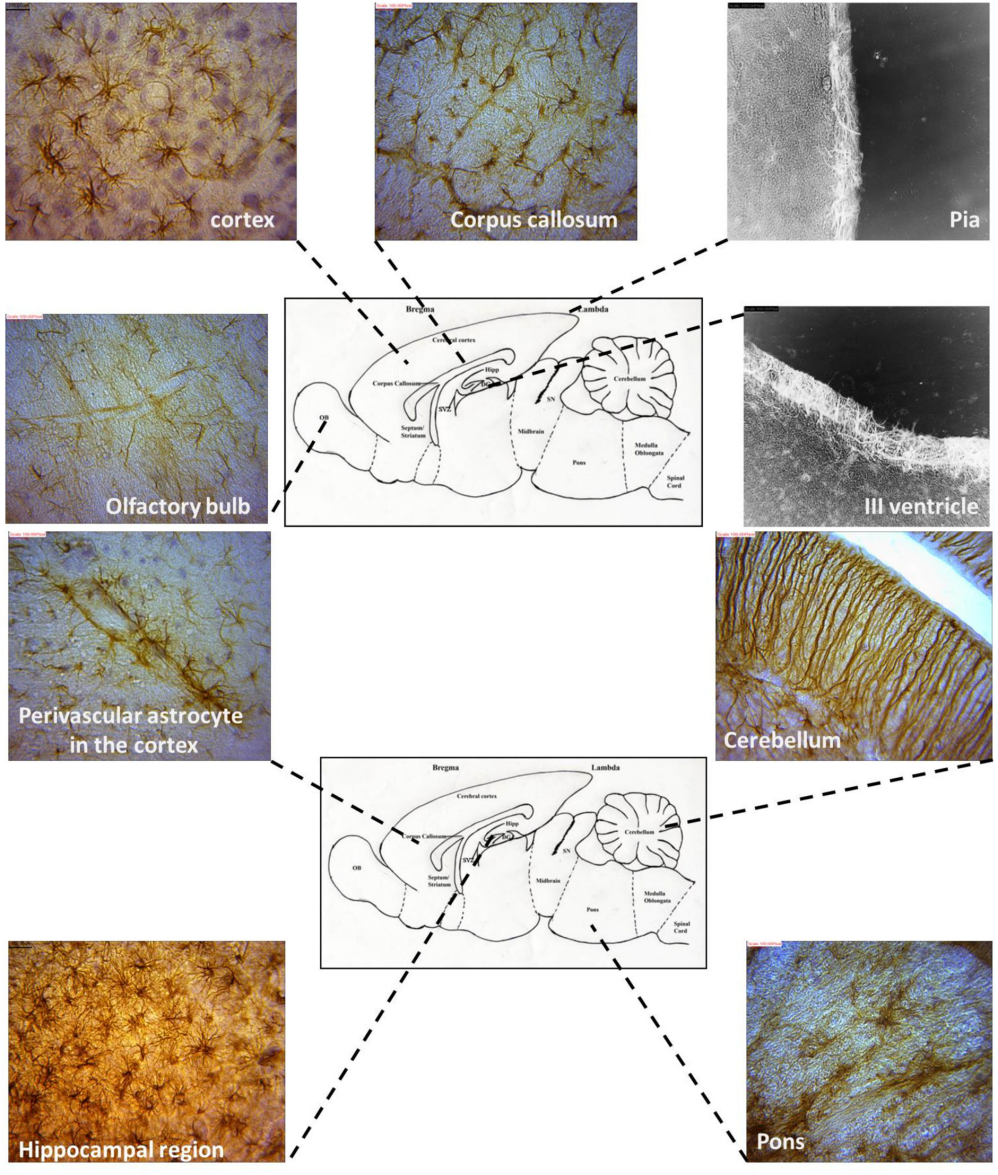
**Representative astrocyte morphologies from selected regions of the AGR brain (Images were obtained from sagittal sections and immunolabeled with GFAP)**.

Astrocyte–Neuron interactions were demonstrated in various brain regions showing terminal neuronal dendritic connections with various astrocytes types. Golgi stain revealed the presence of greater tufts of dendrites in various morphological types of astrocytes than immunohistochemistry. The morphology of astrocytes was also seen to vary throughout the AGR brain using GFAP stain (**Figure 6**).

The location of astrocytes were assessed and analyzed from 12 brain sub regions and revealed eight astrocytic subtypes in the AGR. Distinct regional heterogeneity with respect to the type of astrocytic cell that resides in each neuronally defined region was also observed. Predominantly fibrous astrocytes were found in the corpus callosum and brain stem while protoplasmic astrocytes in the cortex and DG. Both protoplasmic and fibrous astrocytes were stained in hippocampus and OB. Radial glia were found along the OB and SVZ while velate astrocytes were particularly found around populations of granule neurons (cerebellum and hippocampus). Marginal astrocytes were found close to the pia mater. Bergmann glia were found abundantly in the molecular layer of the cerebellum. Perivascular and periventricular astrocytes were identified in the cortex and third ventricle, respectively (**Table 1**; **Figure 6**) and these analyses capture considerable heterogeneity of astrocytic morphologies in the AGR brain. Age group morphological differences were observed with the juvenile sections having more prominent astrocytic processes than in adults.

**Table 1:** Regional heterogeneity of astrocytes in the African giant rats (AGR) brain.

Region	Astrocyte subtypes								
	Radial	Bergmann glia	Protoplasmic	Fibrous	Velate	Marginal	Perivascular	Periventricular
Olfactory bulb (OB)	+		+	+	+/–			
RMS				+				
Cortex			+		+/–		+	
Pial surface				+		+		
Corpus callosum	+			+				
Lateral ventricle/SVZ	+							
Third ventricle	+							+
Hippocampus			+	+	+/–			
DG	+		+					
Cerebellum		+			+/–			
Brainstem				+	+/–			

Astrocyte heterogeneity in juvenile and adult groups revealed eight subtypes to include fibrous astrocytes predominantly in the corpus callosum and brain stem, protoplasmic astrocytes in the cortex and dentate gyrus (DG) and hippocampus; radial glia were found along the olfactory bulb (OB) and subventricular zone (SVZ); velate astrocytes were particularly found in the cerebellum and hippocampus; marginal astrocytes close to the pia mater; Bergmann glia in the molecular layer of the cerebellum; perivascular, and periventricular astrocytes in the cortex and third ventricle, respectively.

### Astrocytic density varies with defined anatomical regions

Estimated cell counts were obtained using 40 μm sagittal sections across the right hemisphere of the AGR brain. Estimated number of cells from five random boxes measuring 1 mm^2^ cells with an average taken. The sum of the astrocyte counts per slide from the mid-sagittal section to the most lateral section was recorded as the total right hemispheral astrocyte count (**Table 2**). The cell population density of astrocytes were found to vary across brain regions and ranged from 1 to 8 cells per mm^2^ in the cortex to 7–17 cells per mm^2^ in the DG. Astrocytic density also varied considerably in sub-regions of the cortex, with 1–5 in layer II–III compared to 1–8 in layer I. In all the brain regions, astrocyte count was higher in juveniles than adults except at the third ventricle. Significant difference in mean counts (*p* ≤ 0.01) between juveniles and adults was found in the cortex, pia, corpus callosum, RMS, DG, molecular, and Purkinje cell layer of the cerebellum. Highest astrocyte counts in both juvenile and adult was found in DG while the least was found in the brain stem and sub cortex, respectively. Age seemed to play a significant role in astrocyte population counts in different regions as there were differences in the numbers of juvenile and adult cell counts per region

**Table 2:** Astrocyte counts in different brain regions.

Brain region	Age	
	Juvenile	Adult
CORTEX^∗^	428.33 ± 70.06	238.33 ± 36.86
PIA^∗^	635.00 ± 185.00	163.33 ± 23.63
CORPUS CALLOSUM^∗^	753.33 ± 83.87	358.33 ± 54.85
HIPPOCAMPUS	580.00 ± 250.60	441.67 ± 68.98
OLFACTORY BULB	255.00 ± 80.47	204.00 ± 31.75
PERIVASCULAR	391.67 ± 143.38	300.00 ± 141.07
RMS^∗^	508.33 ± 30.14	261.67 ± 41.93
SUB CORTEX	206.67 ± 58.60	71.33 ± 8.08
SVZ	555.00 ± 152.23	366.67 ± 56.86
DG^∗^	1110.00 ± 235.16	596.67 ± 80.83
CEREB. GRAN	456.67 ± 60.28	388.33 ± 46.46
CEREB. MOLE^∗^	395.00 ± 30.41	216.67 ± 28.87
CEREB. WM	433.33 ± 51.32	343.33 ± 58.60
CEREB. PCL^∗^	403.33 ± 25.17	306.67 ± 30.55
PONS	280.00 ± 121.24	176.67 ± 20.82
BRAIN STEM	170.00 ± 125.30	143.33 ± 30.55
3^RD^ VENTRICLE	211.67 ± 34.03	213.33 ± 32.15

SVZ, Sub Ventricular Zone; CEREB. GRAN., Granular layer of the Cerebellum; CEREB. MOLE., Molecular layer of Cerebellum; CEREB. WM, Cerebellar White Matter; CEREB. PCL, Purkinje Cell Layer of Cerebellum. Age influences the population of astrocytes in different regions of the brain. In all the brain regions, astrocyte count was higher in juveniles than adults except at the third ventricle. Significant difference in mean counts (^∗^) between juveniles and adults was found in the CORTEX (*t* = 4.16, *p* = 0.01), PIA (*t* = 4.38, *p* = 0.01), CORPUS CALLOSUM (*t* = 6.83, *p* = 0.01), RMS (*t* = 8.27, *p* = 0.01), DG (*t* = 3.58, *p* = 0.01), CEREB. MOLE. (*t* = 7.37, *p* = 0.01) and CEREB. PCL (*t* = 4.23, *p* = 0.01). The highest astrocyte counts in both juvenile and adult was found in DG while the least was found in the BRAIN STEM and SUB CORTEX, respectively.

## Discussion

### GFAP Suitability as Immune Marker

Using the expression of GFAP as prototype marker of astrocytes has been dubbed a myth because of its limitations (Molofsky et al., 2012). GFAP is not an absolute marker of all non-reactive and though detectable in several healthy astrocytes, many mature astrocytes in healthy CNS tissue do not express detectable levels (Sofroniew and Vinters, 2010). Astrocytes exhibit both regional and local variability in GFAP expression regulated by a large number of inter- and intra-cellular signaling molecules (Sofroniew, 2009).

Glial fibrillary acid protein immunohistochemistry does not label all portions of the astrocyte but only in the main stem branches (Sofroniew and Vinters, 2010). GFAP is entirely absent from the finely branching astrocyte processes and is often not detectably present in the cell body (**Figures 1B** vs **5A**). Consequently, GFAP immunohistochemistry can markedly underestimate the extent of astrocyte branching and territory in comparison with other means of detection such as Golgi staining as shown in this study.

We have, however, used anti-GFAP immunohistochemistry to map the morphology, distribution, and densities of astrocytes in the AGR brain across three broad age groups (neonates, juveniles, and adults). It revealed an accumulation of GFAP during early postnatal development in agreement with previous reports (Lewis and Cowan, 1985; Tardy et al., 1989). Landry et al. (1999) suggested that the effect of age in the expression of astrocytes mRNA is a factor that will affect GFAP immunoreactivity intensity. This followed the pattern observed in our present study where neonates had limited expression of GFAP while the highest expressions were found in juveniles with reduced immunoreactivity in the adult group.

From this study, we demonstrated that astrocytic morphology, density and GFAP staining intensity are age dependent in the AGR brain. Astrocytic numbers increase with age by approximately 20% (Rozovsky et al., 1998; Salminen et al., 2011). Two postulates have been raised to explain this: (a) that the increase may be in response to injured or damaged neurons during aging and/or (b) increased astrocytic densities in the aging brain is vital in conferring the same level of neuroprotection that is present in the brain of a young animal. Thus, age, as a factor, should be considered in neuroinflammatory studies (Barreto et al., 2011).

### Morphological Differences

Eight astrocytic subtypes were observed in the brain of the AGR. Several authors have reported on variable subtypes: nine in mice (Emsley and Macklis, 2006; Oberheim et al., 2012), four in humans and primates (Nag, 2011). The cells were in distinct regional heterogeneity with respect to the type of astrocytic cell that resides in each neuronally defined region of the adult CNS (**Figure 6**). The distinct, region-specific heterogeneity of astrocytes observed in the AGR is, thus, comparable to the regionalization and heterogeneity of defined neuronal subtypes (Emsley and Macklis, 2006). Assessing 12 CNS sub-regions, we found, for example, predominantly velate, protoplasmic, fibrous, and radial astrocytes. There were also differences in the morphological appearances across age groups which might relate functional significance to morphology in AGR astrocytes (Oberheim et al., 2012).

### Astrocyte Morphology Affects Neural Function?

Existing literature suggests that the degree of varied astrocytic sub-types morphology and cellular density seen depends on how astrocytes are labeled and thus, other stains and techniques should be carried out in future analysis to differentiate or further elucidate the morphology and cell density (Emsley and Macklis, 2006).

However, other authors indicate that various morphological sub-types exhibit varying structural and functional properties that might be involved in neural function of the particular brain regions, e.g., in the Cerebellum (Wang et al., 2011), Hippocampus (D’Ambrosio et al., 1998), OB (Olude et al., 2014b). Although the signaling pathway of these modifications are yet unknown. Baseline data on morphology and density of astrocytes in brains of mammals across age groups can thereby serve as a tool to predict or detect any functional correlate between normal decline in cellular density with aging against functional depletion of cells and their synaptic relevance in astrocytic diseases.

Regional density has been reported (Emsley and Macklis, 2006). The AGR also showed regional density but the cell populations differ from their reports. This might be due to the GFAP antibody signaling power, a slightly thicker section utilized in this study or generally a morphological finding in the AGR as having bigger astrocytic cells and processes. The cell bodies span over 10 mm in some regions and processes up to 90 mm. This may account for an increase in volume than in the laboratory rodent suggesting that density alone does not account for astrocytic function but synaptic volume (Şovrea and Boşca, 2013). That age groups present increasing number of astrocytic populations indicates that the functional significance of astrocytes will progressively increase from neonates to juvenile and subsequently be reduced in the adults. The significance of some morphological types and densities being depleted with age is unknown but might account for certain diseases or tumors if their levels go beyond normal. Thus, it is important to establish normal baseline data for morphological subtypes with their regional densities in order to map functionally significant levels as possible indicators of astrocytic or neuroinflammatory diseases.

This study has shown baseline data of astrocyte sub types and their estimated densities in different regions of the AGR brain. This offers great pilot value for cell culture studies in the AGR brain because of its indigenous nature, behavioral qualities such as olfaction, memory, and locomotor abilities which will be useful in studying nervous conditions that involve astrocytes from an African perspective.

## Conclusion

The authors note that there are distinct and region-specific differences in astrocytic morphology in the AGR brain. These morphologic differences are as diverse as those for neuronal populations across the adult mammalian CNS, and can be used to define anatomical regions. Astrocytic density varies considerably, and can distinctly and independently define neuronally defined anatomical regions and sub-regions. Authors propose that specific astrocyte populations perform specific roles in different brain regions. Case in point is the radial glia and its possible roles in olfaction in the AGR. This study therefore, demonstrates that astrocytic morphology and density independently define the discrete cytoarchitecture of the AGR brain and presents the juvenile AGR as a suitable research model for astrocytic studies.

## Author Contributions

All listed authors (MO, OM, OA, JO, AI) met the four underlisted criteria. They:

• made substantial contributions to the conception and/or design of the work; the acquisition, analysis, and interpretation of data of this study;
• were involved in the drafting the work and/or revising it critically for important intellectual content;
• gave final approval of the version of the manuscript submitted to be published; and
• all agree to be accountable for all aspects of the work in ensuring that questions related to the accuracy or integrity of any part of the work are appropriately investigated and resolved.

## Conflict of Interest Statement

The authors declare that the research was conducted in the absence of any commercial or financial relationships that could be construed as a potential conflict of interest.

## References

[B1] Ajayi S. S. (1974). *The Biology and Domestication of African Giant Rat (Cricetomys gambianus Waterhouse)*. Ph.D. thesis, University of Ibadan Ibadan.

[B2] Allen N. J. Chakraborty C. Howe M. L. Barres B. A. (2011). “Identification of an astrocyte-derived factor that promotes the formation of excitatory synapses containing GluA1 AMPA glutamate receptors,” in *Neuroscience Meeting Planner, Program No. 436.10/A61* (Washington, DC: Society for Neuroscience).

[B3] Barres B. A. (2008). The mystery and magic of glia: a perspective on their roles in health and disease. *Neuron* 60 430–440. 10.1016/j.neuron.2008.10.013 18995817

[B4] Barreto G. E. Gonzalez J. Capani F. Morales L. (2011). “Role of astrocytes in neurodegenerative diseases,” in *Neurodegenerative Diseases – Processes, Prevention, Protection and Monitoring* Chap. 11 ed. Chang R. C.-C. (Rijeka: InTech) 257–272. Available at: http://www.intechopen.com/books/neurodegenerative-diseasesprocesses-prevention-protection-andmonitoring/role-of-astrocytes-in-neurodegenerative-diseases

[B5] Batter D. K. Corpina R. A. Roy C. Spray D. C. Hertzberg E. L. Kessler J. A. (1992). Heterogenicity in gap junction expression in astrocytes cultured from different brain regions. *Glia* 6 213–221. 10.1002/glia.440060309 1282501

[B6] Christopherson K. S. Ullian E. M. Strokes C. C. Mullowney C. E. Hell J. W. Agah A. (2005). Thrombospondins are astrocyte- secreted proteins that promote CNS synaptogenesis. *Cell* 120 421–433. 10.1016/j.cell.2004.12.020 15707899

[B7] D’Ambrosio R. Wenzel J. Schwartzkroin P. A. McKhann G. M. Janigro D. (1998). Functional specialization and topographic segregation of hippocampal astrocytes. *J. Neurosci.* 18 4425–4438. 9614220 10.1523/JNEUROSCI.18-12-04425.1998 PMC4093786

[B8] Emsley J. G. Arlotta P. Macklis J. D. (2004). Star-crossed neurons: astroglial effects on neural repair in the adult mammalian CNS. *Trends Neurosci.* 27 238–240. 10.1016/j.tins.2004.02.008 15111002

[B9] Emsley J. G. Macklis J. D. (2006). Astroglial heterogeneity closely reflects the neuronal-defined anatomy of the adult murine CNS. *Neuron Glia Biol.* 2 175–186. 10.1017/S1740925X06000202 17356684 PMC1820889

[B10] Gabriel S. Njunting M. Pomper J. K. Merschhemke M. Sanabria ER Eilers A (2004). Stimulus and potassium-induced epileptiform activity in the human dentate gyrus from patients with and without hippocampal sclerosis. *J. Neurosci.* 24 10416–10430. 10.1523/JNEUROSCI.2074-04.2004 15548657 PMC6730304

[B11] Hewett J. A. (2009). Determinants of regional and local diversity within the astroglial lineage of the normal central nervous system. *J. Neurochem.* 110 1717–1736. 10.1111/j.1471-4159.2009.06288.x 19627442

[B12] Ishibashi T. Dakin K. A. Stevens B. Lee P. R. Kozlov S. V. Stewart C. L. (2006). Astrocytes promote myelination in response to electrical impulses. *Neuron* 49 823–832. 10.1016/j.neuron.2006.02.006 16543131 PMC1474838

[B13] Kálmán M. Hajos F. (1989). Distribution of glial fibrillary acidic protein (GFAP)-immunoreactive astrocytes in the rat brain. *Exp. Brain Res.* 78 147–163. 10.1007/BF00230694 2591509

[B14] Kettenmann H. K. Ransom B. R. (2005). *The Concept of Neuroglia: A Historical Perspective in Neuroglia*. Oxford: Oxford University Press 1–16.

[B15] Kimelberg H. K. (2004). The problem of astrocyte identity. *Neurochem. Int.* 45 191–202. 10.1016/j.neuint.2003.08.015 15145537

[B16] Koehler R. C. Roman R. J. Harder D. R. (2009). Astrocytes and the regulation of cerebral blood flow. *Trends Neurosci.* 32 160–169. 10.1016/j.tins.2008.11.005 19162338

[B17] Kosaka T. Kosaka K. (2009). “Olfactory bulb anatomy,” in *Encyclopedia of Neuroscience* 7th Edn ed. Squire L. R. (Oxford University Press) 59–69. 10.1016/B978-008045046-9.01686-7

[B18] Landry C. F. Pribyl T. M. Ellison J. A. Givogri M. I. Kampf K. Campagnoni C. W. (1999). Embryonic expression of the basic protein gene – identification of a promoter region that targets transgene expression to pioneer neurons. *J. Neurosci.* 18 7315–7327. 9736652 10.1523/JNEUROSCI.18-18-07315.1998 PMC6793259

[B19] Lewis S. A. Cowan N. J. (1985). Temporal expression of mouse glial fibrillary acidic protein mRNA studied by a rapid in situ hybridization procedure. *J. Neurochem.* 45 913–919. 10.1111/j.1471-4159.1985.tb04080.x 2411859

[B20] Lutz S. E. Zhao Y. Gulinello M. Lee S. C. Raine C. S. Brosnan C. F. (2009). Deletion of astrocyte connexins 43 and 30 leads to a dysmyelinating phenotype and hippocampal CA1 vacuolation. *J. Neurosci.* 29 7743–7752. 10.1523/JNEUROSCI.0341-09.2009 19535586 PMC2737812

[B21] Molofsky A. V. Krenick R. Ullian E. Tsai H. Deneen B. Richardson W. D. (2012). Astrocytes and disease: a neurodevelopmental perspective. *Genes Dev.* 26 891–907. 10.1101/gad.188326.112 22549954 PMC3347787

[B22] Mrzljak L. Uylings H. B. M. Van Eden G. G. Judáš M. (1991). “Neuronal development in human prefrontal cortex in prenatal and postnatal stages,” in *Progress in Brain Research* Chap. 9 Vol. 85 eds Uylings H. B. M. van Eden C. G. de Bruin J. P. C. Corner M. A. Feenstra M. G. P. (Amsterdam: Elsevier) 85 185–222. 10.1016/s0079-6123(08)62681-3 2094894

[B23] Nag S. (2011). Morphology and properties of brain endothelial cells. *Methods Mol. Biol.* 686 3–47. 10.1007/978-1-60761-938-3_1 21082365

[B24] Nedergaard M. Verkhratsky A. (2012). Artifact versus reality – how astrocytes contribute to synaptic events. *Glia* 60 1013–1023. 10.1002/glia.22288 22228580 PMC3340515

[B25] Newman E. A. (2003). Glial cell inhibition of neurons by release of ATP. *J. Neurosci.* 23 1659–1666. 12629170 10.1523/JNEUROSCI.23-05-01659.2003 PMC2322877

[B26] Oberheim N. A. Goldman S. A. Nedergaard M. (2012). Heterogeneity of astrocytic form and function. *Methods Mol. Biol.* 814 23–45. 10.1007/978-1-61779-452-0_3 22144298 PMC3506190

[B27] Olude M. A. (2014). Neurocellular profile in the brain of the African giant rat (*Cricetomys gambianus*, Waterhouse). Ph.D. thesis, University of Ibadan Ibadan.

[B28] Olude M. A. Olopade J. O. Ihunwo A. O. (2014a). Adult neurogenesis in the African giant rat (*Cricetomys gambianus*, Waterhouse 1840). *Metab. Brain Dis.* 29 857–866. 10.1007/s11011-014-9512-9 24577632

[B29] Olude M. A. Ogunbunmi T. K. Olopade J. O. Ihunwo A. O. (2014b). The olfactory bulb structure of African giant rat (*Cricetomys gambianus*, Waterhouse 1840) I: cytoarchitecture. *Anat. Sci. Int.* 89 224–231. 10.1007/s12565-014-0227-0 24469950

[B30] Ota Y. Zanetti A. T. Hallock R. M. (2013). The role of astrocytes in the regulation of synaptic plasticity and memory formation. *Neural Plast.* 2013 185463 10.1155/2013/185463 PMC3867861 24369508

[B31] Panov A. Orynbayeva Z. Vavilin V. Lyakhovich V. (2014). Fatty acids in energy metabolism of the central nervous system. *Biomed Res. Int.* 2014 472459 10.1155/2014/472459 PMC4026875 24883315

[B32] Quaegebeur A. Lange C. Carmeliet P. (2011). The neurovascular link in health and disease: molecular mechanisms and therapeutic implications. *Neuron* 71 406–424. 10.1016/j.neuron.2011.07.013 21835339

[B33] Ramón y Cajal S. (1909). *Histologie du Systeme Nerveux del’Homme et Des Vertebres*. (Paris: Editions Maloine).

[B34] Rozovsky I. Finch C. E. Morgan T. E. (1998). Age-related activation of microglia and astrocytes: in vitro studies show persistent phenotypes of aging, increased proliferation, and resistance to down-regulation. *Neurobiol. Aging* 19 97–103. 10.1016/S0197-4580(97)00169-3 9562510

[B35] Salminen A. Ojala J. Kaarniranta K. Haapasalo A. Hiltunen M. Soininen H. (2011). Astrocytes in the aging brain express characteristics of senescence-associated secretory phenotype. *Eur. J. Neurosci.* 34 3–11. 10.1111/j.1460-9568.2011.07738.x 21649759

[B36] Sofroniew M. V. (2009). Molecular dissection of reactive astrogliosis and glial scar formation. *Trends Neurosci.* 32 638–647. 10.1016/j.tins.2009.08.002 19782411 PMC2787735

[B37] Sofroniew M. V. Vinters H. V. (2010). Astrocytes: biology and pathology. *Acta Neuropathol.* 119 7–35. 10.1007/s00401-009-0619-8 20012068 PMC2799634

[B38] Şovrea A. S. Boşca A. B. (2013). Astrocytes reassessment – an evolving concept part one: embryology, biology, morphology and reactivity. *J. Mol. Psychiatry* 1 18 10.1186/2049-9256-1-18 PMC4445578 26019866

[B39] Stevens B. Allen N. J. Vazquez L. E. Howell G. R. Christopherson K. S. Nouri N. (2007). The classical complement cascade mediates CNS synapse elimination. *Cell* 131 1164–1178. 10.1016/j.cell.2007.10.036 18083105

[B40] Takano T. Tian G. F. Peng W. Lou N. Libionka W. Han X. (2006). Astrocyte-mediated control of cerebral blood flow. *Nat. Neurosci.* 9 260–267. 10.1038/nn1623 16388306

[B41] Tardy M. Fages H. Riol G. LePrince P. Rataboul C. Charriere-Bertrand C. (1989). Developmental expression of glial fibrillary acidic protein mRNA in the central nervous system and in cultured astrocytes. *J. Neurochem.* 52 162–167. 10.1111/j.1471-4159.1989.tb10911.x 2908887

[B42] Venance L. Prémont J. Glowinski J. Giaume C. (1998). Gap junctional communication and pharmacological heterogeneity in astrocytes cultured from the rat striatum. *J. Physiol. (Lond.)* 510 429–440. 10.1111/j.1469-7793.1998.429bk.x 9705994 PMC2231053

[B43] Verkhratsky A. Kettenman H. (1996). Calcium signaling in glial cells. *Trends Neurosci.* 19 346–352. 10.1016/0166-2236(96)10048-5 8843604

[B44] von Bohlen Halbach O. (2011). Immunohistological markers for proliferative events, gliogenesis and neurogenesis within the adult hippocampus. *Cell Tissue Res.* 345 1–19. 10.1007/s00441-011-1196-4 21647561

[B45] Wang X. Imura T. Sofroniew M. V. Fushiki S. (2011). Loss of adenomatous polyposis coli in Bergmann glia disrupts their unique architecture and leads to cell non-autonomous neurodegeneration of cerebellar Purkinje neurons. *Glia* 59 857–868. 10.1002/glia.21154 21381115 PMC3287075

[B46] Weetjens B. J. Mgode G. F. Machang’u R. S. Kazwala R. Mfinanga G. Lwilla F. (2009). African pouched rats for the detection of pulmonary tuberculosis. *Int. J. Tuberc. Lung Dis.* 13 737–743. 19460250

[B47] Zhang C. Hua T. Zhu Z. Luo X. (2006). Age-related changes of structures in cerebellar cortex of cat; *J. Biosci.* 31 55–60. 10.1007/BF02705235 16595875

